# Important Health Care Components to Promote Sexual and Reproductive Health Among Young Women With Attention‐Deficit/Hyperactivity Disorder: A Delphi Study

**DOI:** 10.1111/hex.70856

**Published:** 2026-09-02

**Authors:** Karin Wallin, Lena Hanberger, Inger Wallin‐Lundell, Siw Alehagen, Sally Hultsjö

**Affiliations:** ^1^ Department of Obstetrics and Gynecology in Linköping Linköping University Linköping Sweden; ^2^ Department of Health, Medicine and Caring Sciences Linköping University Linköping Sweden; ^3^ Department of Health Sciences Swedish Red Cross University Huddinge Sweden; ^4^ Department of Psychiatry Ryhov County Hospital Jönköping Sweden

**Keywords:** ADHD, Delphi study, health care professionals, health promotion, sexual and reproductive health, young women

## Abstract

**Introduction and Objectives:**

Living with attention‐deficit/hyperactivity disorder (ADHD) may negatively affect young women's sexual and reproductive health (SRH) if they lack adequate support. To promote positive and safe sexual and relational experiences, this study aimed to identify important components within health care settings that can promote SRH among young women with ADHD.

**Methods:**

An expert panel of young women (aged 19–29 years) with ADHD and health care professionals (HCPs) working in SRH counselling or psychiatric clinics rated the importance of statements on the topic across two survey rounds.

**Results:**

Consensus was reached on about two‐thirds of the statements. High agreement emphasized the need for HCP training on ADHD and SRH in both psychiatric clinics and SRH clinics, including knowledge of hormonal influences. Important components also included routine inquiries about sexuality and relationships, and sexual violence; accessible health care; inclusive SRH information that acknowledges variation of symptoms and functional abilities; discussion groups on sexuality and relationships for young women; and integrating SRH in existing psychoeducation.

**Conclusion:**

SRH among young women with ADHD may benefit from several health care efforts delivered across both sexual health and psychiatric services, including accessible care tailored to their needs, structured clinical routines and appropriate training in SRH and ADHD for HCPs.

**Patient and Public Contribution:**

Young women with experience of living with ADHD and HCPs with varied clinical expertise participated in the Delphi study. Women with ADHD were involved as co‐researchers in the survey development and in the pilot‐testing.

## 1 Introduction

Sexual and reproductive health (SRH) significantly impacts overall health and well‐being [1, 2]. Autonomy in making informed and responsible decisions, free from coercion, discrimination or violence, is essential for SRH and is considered a fundamental human right [1, 2]. For young women with ADHD, as with all young women, an increased desire and engagement in sexual and relational experiences are normative and crucial for understanding their own sexuality and developing relational skills. Sexuality and relationships are shaped by personal traits and values, influenced by social contexts, such as interactions with peers, parents and school, as well as broader social and cultural norms [3, 4]. Symptoms of ADHD that affect social interactions and behaviours in daily life, fluctuations of ADHD symptoms during the menstrual cycle and perceived expectations regarding sexual behaviours and relationships may pose challenges for young women with ADHD and their SRH [5, 6, 7, 8]. Therefore, it is crucial for healthcare services to provide a comprehensive and evidence‐based strategy to promote positive sexual experiences and healthy relationships in young women with ADHD.

According to an online survey, young people with ADHD were associated with a more varied approach to sexual interests and practices compared to their neurotypical peers. This included a greater likelihood of trying different sexual activities, earlier initiation of first sexual intercourse, and more frequent engagement in same‐sex or bisexual experiences [9]. A qualitative study further associated fearlessness in forming relationships and curiosity about exploring their sexuality could lead to positive sexual experiences and a deeper understanding of sexuality among young women [8]. However, young women with ADHD tend to report lower satisfaction with both their sexual lives and their romantic relationships compared to neurotypical peers. While emotion dysregulation and conflict have been shown to negatively affect relationship quality, impulsivity and inattention has been associated with sexual dysfunction [9, 10, 11, 12]. Impulsiveness and inattention in young women with ADHD have also been linked to a higher likelihood of engaging in casual sexual relationships, not using condoms, and experiencing sexual violence [9, 13, 14, 15, 16]. Consequently, they face a higher risk of contracting sexually transmitted infections (STIs) and becoming pregnant at an early age [17, 18].

Secondary outcomes of living with ADHD may further affect SRH. Gender differences in symptom presentation may delay ADHD assessment and diagnosis, resulting in missed opportunities for early intervention and health care support [19]. Young women with ADHD often experience low self‐esteem due to early experiences of peer rejection and feelings of inadequacy [20, 21]. This may hinder them from communicating their wishes and desires in sexual situations and relationships, fearing rejection by a partner [6, 8]. It has also been suggested that lower peer acceptance and vulnerability to peer rejection can motivate risk‐taking and coercion into sexual activities, including online sexual exploitation [19, 22, 23]. Common comorbid conditions in young women with ADHD, such as anxiety and depression, can further negatively impact sexual interest and function [24, 25, 26].

In Sweden, young people can receive free counselling on SRH and access STI testing or be referred to testing through public youth clinics, school health clinics and sexual health clinics. These clinics are primarily run by school nurses, midwives, gynaecologists, counsellors and psychologists [27, 28]. While these clinics also offer mental health counselling, psychiatric outpatient clinics within public healthcare are mainly responsible for ADHD assessment and treatment. Psychoeducational interventions, medical treatment and counselling are provided by psychiatric nurses, occupational therapists, counsellors, psychologists and psychiatrists [29]. Evidence‐based informational websites, funded by the Swedish government, further complement physical clinics by providing accessible health information, guidance and digital support on SRH and general health issues [30, 31].

To ensure young women with ADHD have safe and positive sexual experiences and relationships, both symptoms and contextual factors need to be taken into consideration when promoting SRH in clinical settings. Providing person‐centred care, taking personal preferences and needs into account, is crucial as ADHD symptoms, functionality and context vary widely within the group [7, 32]. Additionally, health care professionals (HCPs) must ensure non‐discriminatory encounters as young women with ADHD may experience stigma and fear of judgement in clinical settings [33], particularly as engaging in sexual risk‐taking behaviours may be considered to break social expectations on female sexuality [19]. Previous studies have emphasized the importance of early interventions directed at young women with ADHD and SRH and the need for accessible health facilities [17, 34, 35].

Although previous studies on SRH among young women with ADHD suggest several supportive actions, comprehensive clinical guidelines specifically targeting this group remain limited [6, 9, 11, 36]. To improve SRH outcomes at the individual level, health‐care services must understand which structures, routines and professional competencies are most important for young women with ADHD. A structured consensus process with input from women with lived experience, as well as from those who deliver care, could help determine which components should be prioritized and incorporated into evidence‐based support [37]. Achieving such consensus can guide the development of support that promotes safe and positive sexual and relational experiences, reduces vulnerability to risk‐taking and ensures accessible and equitable services. The findings of this study may contribute to the development of clinical guidelines and routines that address the specific needs and challenges of young women with ADHD, in line with the United Nations’ global commitment to equitable and inclusive health care for all populations [38]. Including both young women with ADHD and HCPs working in SRH and/or psychiatry may strengthen the relevance and applicability of the results, as end‐user engagement increases the usefulness of clinical recommendations [39]. Involving HCPs from different clinical areas can also help highlight forms of support that reflect a broader context, as previous research shows that addressing issues related to SRH may not be restricted only to sexual‐health clinics [40].

Thus, the aim was to identify important components within health care settings that can promote SRH among young women with ADHD.

## 2 Methods

### 2.1 Delphi Technique

**Figure 1:**
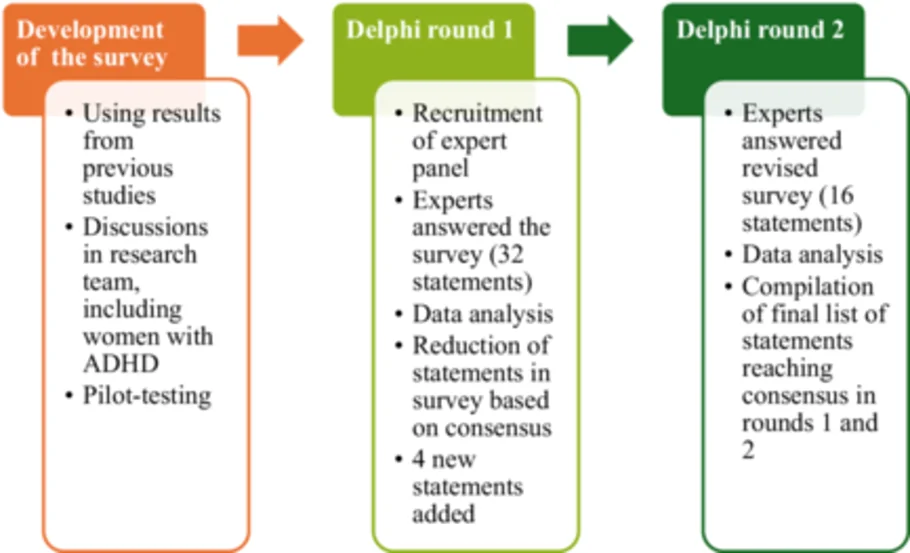
Process of the study based on the Delphi technique.

The Delphi technique was chosen because it is well suited for achieving consensus on complex issues, particularly when empirical evidence is limited and the target population has diverse experience and expertise [41]. For this study, the Delphi technique provided an opportunity to acknowledge and prioritize important health care components that may improve SRH outcomes, drawing on the lived experiences of young women with ADHD and the perspectives of HCPs. The Delphi technique is a structured group communication method designed to gather the knowledge and opinions of experts [37]. The method involves an iterative process of multiple rounds of data collection where feedback is provided after each round, allowing experts to anonymously refine their responses based on the collective input of the group (Figure 1) [37]. This study included statement generation and two rounds of online surveys. The expert panel consisted of young women with ADHD and HCPs working within youth health, school health, adult sexual health and psychiatry (Tables 1 and 2). The use of an online survey was justified by its ability to ensure anonymity and to enable participation from a geographically dispersed group of diverse stakeholders [42]. The number of rounds was determined in advance. Given previous experiences within the research group of challenges in recruiting young women with ADHD, two rounds were chosen to reduce the risk of participant fatigue and dropout [43].

**Table 1:** Characteristics of participating women.

Variables	*n* = 27
Age	
19–23	10
24–29	17
Median	26
Level of education	
Elementary school	8
Secondary school	13
Higher vocational education	1
University	5
Occupation	
Work	12
Student, secondary school	3
Student, university	9
Sick leave	2
No response	1
Age at time of ADHD diagnosis	
10–18	11
19–28	16
Median	20
Type of neuropsychiatric diagnoses	
ADHD‐combined type	19
ADHD‐predominantly inattentive type	8
Current psychiatric diagnoses and other neuropsychiatric diagnoses	
Autism spectrum disorder	3
Depression	2
Depression and anxiety	2

**Table 2:** Characteristics of participating health care professionals.

Variables	*n* = 36
Age	
33–49	18
50–66	18
Median	50
Years in profession	
3–14	17
15–37	17
Median	15
Gender	
Men	5
Women	31
Clinical speciality	
School health	6
Psychiatric health	16
Sexual health (adult)	8
Youth health	6
Profession	
Counsellor	7
Gynaecologist	1
Midwife	6
Occupational therapist	3
Physiotherapist	1
Psychiatric nurse	4
Psychiatric nursing assistant	2
Psychiatrist	2
Psychotherapist	4
School nurse	6

### 2.2 Development of the Survey

Surveys in a Delphi study may be developed from existing literature, prior empirical work or established guidelines [44]. Given the limited knowledge in the studied topic, qualitative studies focusing on experiences and perceptions of SRH among young women with ADHD, and related support needs, were conducted in the research group prior to this study [8, 40, 45]. The statements in the present study were primarily derived from this work and were selected based on the content described in the main themes and categories identified in the analyses of the included studies. Because these studies did not specifically examine the impact of hormonal fluctuations on SRH in young women with ADHD, additional statements were developed to address this aspect [46, 47].

An initial survey draft was developed by the research group through collaborative discussions. To ensure the survey was clear, relevant and usable for the target group, research partners with lived experience of ADHD or clinical experience were then involved [48]. Initially, two women with ADHD were recruited as research partners via a patient research group. The women first reviewed each statement independently and provided comments on adequacy, clarity and non‐stigmatizing wording. Their feedback was then discussed jointly with research team members, and statements were revised accordingly. Any disagreements were resolved through discussions. The survey was further updated based on the women's suggestions for additional statements and feedback on the overall survey design. The revised version was then pilot tested by the women, along with one midwife and one psychologist. Some statements were rephrased, and the survey was shortened to make it more manageable for young women with ADHD. The final version contained 32 statements distributed in the first round. Each statement was rated on a four‐point scale ranging from “do not agree at all” to “fully agree” and participants had the option to provide written comments for each statement. The survey was developed and distributed using the data platform Artologik Survey and Report. To reduce the risk of automated or invalid responses, the dataset was screened prior to analysis for duplicate entries, atypical response patterns and implausibly short completion times.

### 2.3 Sampling and Recruitment

Recruitment in Delphi studies is based on individuals who are believed to have expertise in the investigated subject [37]. According to Boulkedid et al. [42], the credibility and acceptance of the outcome could be enhanced by a heterogeneous expert panel, representing the entire spectrum of stakeholders. To capture diverse experiences and opinions on how to promote SRH, the inclusion criteria for recruitment identifying as a woman (aged 15–29) and having ADHD or being an HCP working in the fields of SRH or psychiatry. The chosen age range represents the period from sexual debut to the onset of common family formation, characterized by sexual and relational exploration [3]. In Sweden, caregiver consent is not required for youths aged 15, provided they receive sufficient information ([49] concerning the ethical review of research involving humans). Clinics were chosen to represent health care settings where young women with ADHD were likely to attend. HCPs were recruited at seven outpatient units belonging to adult psychiatric clinics at three different hospitals. Furthermore, three youth clinics in primary care, one private sexual health clinic for adults and school nurses from six public high schools in one town also participated. The clinics and schools were situated in 10 small and medium‐sized cities and one large city in southeastern and central Sweden. In addition to the clinics and schools that participated, two psychiatric clinics specializing in paediatrics and the authority in charge of high school health services in one more city were approached, but chose not to participate or did not reply. HCPs at participating clinics received information and a weblink to the first survey via e‐mail. To reach the women, information about the study and a weblink to the first survey were disseminated through Facebook pages belonging to a national interest association for people with ADHD and a patient resource group with experience of psychiatric illness. The women were given written information on how to obtain the study information, and the survey was also available at the same clinics where the HCPs were recruited. Women and HCPs who wished to participate had to give their consent via the weblink in order to access the statements in the survey. A total of 27 women aged 19–29 years and 36 HCPs consented to participate in two Delphi rounds. A previous study concluded that 20 to 30 experts answering all statements within each subgroup (e.g., women or HCPs) is enough to ensure moderate replicability of the study [50]. The sample size was therefore assumed to be large enough for the purpose of the study and to account for potential participant dropout. No incentives were offered for participation.

### 2.4 Data Collection

The survey weblink was available from November 2024 to March 2025. The results from the first round were analyzed to identify which statements had reached consensus. The survey was then revised to include the 12 statements that had not reached consensus. The revised survey also included feedback on how the expert group rated those statements in the first round. Written comments from the expert group (women and HCPs) were systematically reviewed to identify new statements and potential ambiguities. Similar comments were then grouped, leading to the addition of four new statements related to the accessibility and availability of health care and information, and one concerning the provision of person‐centred information. Two of the original statements were also rephrased because they were considered difficult to understand. The revised survey, including 16 statements in total, was then sent back to the experts for a second round of ratings. All 63 experts answered the survey in the first round, while 23 women and 31 HCPs completed the second round. Several reminders were sent out.

### 2.5 Data Analysis

In Delphi studies, there is no general agreement on consensus level; however, the level and type of consensus should be defined in advance [37]. Consensus levels commonly used tend to be based on a predefined percentage of agreement, central tendencies or a combination of both [44]. In this study, two criteria had to be fulfilled to reach consensus. First, at least 50% of the experts had to give the same ranking to a statement. Second, the statement also had to reach at least 80% agreement, leaning either toward the positive side (largely agree or fully agree) or to the negative side (do not agree at all or partially agree). This combined approach and the selected consensus levels were based on a previous Delphi study by Hultsjö et al. [51], suggesting that this approach may strengthen the robustness of consensus by reducing dispersion in responses while ensuring clear directional agreement. The results from the participating experts were analyzed with descriptive statistics using IBM SPSS Statistics version 29.0 and presented as frequencies and percentages. The data analysis was performed both on the full expert group panel and separately for young women and HCPs, with equal weighting applied to each participant.

## 3 Results

Approximately two‐thirds of the statements were identified as important components promoting SRH in the full expert panel, reaching consensus in the first round. These statements all leaned toward the positive side (largely or fully agree) (Table 3). Statements reaching consensus concerned five areas: the conduct of HCPs, accessibility and availability to health care, information and education, contraceptive counselling and the menstrual cycle. Some of the statements reached a higher level of agreement among the expert group (above 80% fully agreed). These included the importance of being treated without prejudice and not feeling judged in meetings with HCPs, the need for HCPs in both psychiatric and sexual health clinics to receive training on ADHD, the responsibility of HCPs to assist in contacting other clinics if they cannot provide the necessary support and the importance of HCPs being aware of how the menstrual cycle can affect ADHD symptoms, including the possibility that severe premenstrual syndrome (PMS) may be more common among women with ADHD. Statements with a more moderate unanimity concerned the importance of HCPs to not only discuss the risks of sex but also highlight the positive aspects, where and how information on how sexuality and relationship may be influenced by ADHD should be accessible, which type of structure could help in contraceptive counselling, and whether sexuality and relationships should be included in psychoeducation provided within psychiatry to people with ADHD. Statements with more moderate unanimity concerned the need for HCPs to discuss both risks and positive aspects of sexuality, the accessibility and format of targeted information on sexuality and relationships, structures to support contraceptive counselling, youth clinic accessibility and whether sexuality and relationships should be included in psychiatric psychoeducation.

**Table 3:** Statements reaching consensus in the first round. *N* = 63, young women with attention deficit/hyperactivity disorder (ADHD) and health care professionals.

	Do not agree at all (%)	Partially agree (%)	Largely agree (%)	Fully agree (%)
*Conduct of health care professionals*				
It is important to be treated without prejudice and not feel judged in meetings with health care professionals.	0	4.7	3.2	92.1
It is important that health care professionals not only discuss the risks of sex but also highlight the positive aspects.	1.6	7.9	27.0	63.5
*Information and education*				
Health care professionals in psychiatry should receive training on how women with ADHD can experience sexuality and relationships.	0	6.4	6.5	87.1
Healthcare professionals at sexual health clinics* should receive training about ADHD (*youth clinics, school health, gynaecological clinics, midwifery clinics, private sexual health clinics).	0	5.0	9.8	85.2
There should be written information on 1177.se (national healthcare guidance) describing how sexuality and relationships can be experienced by people with ADHD.	1.6	3.3	19.7	75.4
Sexuality and relationships should be included in psychoeducation provided within psychiatry to people with ADHD.	0	8.1	17.7	74.2
There should be written information at sexual health clinics* describing how sexuality and relationships can be experienced by people with ADHD (*youth clinics, school health services, gynaecological clinics, midwifery clinics, private sexual health clinics).	0	5.0	25.0	70.0
There should be video links on 1177.se that address how sexuality and relationships can be experienced by people with ADHD.	4.9	9.9	26.2	59.0
*Accessibility and availability of health care*				
It is important that health care professionals help me contact other clinics if they cannot provide the support I need.	0	4.9	14.5	80.6
There should be drop‐in times at youth clinics.	1.6	4.9	17.7	75.8
Youth clinics should offer the possibility of digital meetings.	0	8.1	27.4	64.5
It should be possible to contact health care services anonymously for advice on sexuality and relationships.	3.2	4.8	34.9	57.1
Youth clinics should have evening hours.	0	16.1	32.3	51.6
*Contraceptive counselling*				
During contraceptive counselling, it can be helpful to receive written information.	1.6	1.6	21.0	75.8
During contraceptive counselling, it can be helpful to receive a summary of what was discussed.	1.6	6.4	21.0	71.0
During contraceptive counselling, it can be helpful to have verbal information repeated.	3.2	6.5	27.4	62.9
During contraceptive counselling, it can be helpful to know in advance what the conversation will include.	3.2	9.7	24.2	62.9
*The menstrual cycle*				
Health care professionals should be aware that women can have more symptoms of ADHD during certain parts of the menstrual cycle.	1.6	0	8.2	90.2
Health care professionals should be aware that severe PMS (premenstrual syndrome; physical, emotional, and behavioural problems before menstruation) is more common in women with ADHD.	1.6	1.7	8.2	88.5
When ADHD medication is prescribed or followed up on, health care professionals should ask how the menstrual cycle affects my ADHD symptoms.	4.8	6.5	11.3	77.4

In the second round, consensus was reached for another eight statements in the expert group. All statements leaned towards the positive side. Important components were identified in the areas of conduct of HCPs, information and education and contraceptive counselling (Table 4). In the expert group, 87% fully agreed on the newly added statement that information about how sexuality and relationships may be experienced by people with ADHD should emphasize that each individual has unique needs and resources.

**Table 4:** Statements reaching consensus in round 2. *N* = 54, young women with attention deficit/hyperactivity disorder (ADHD) and health care professionals.

	Do not agree at all (%)	Partially agree (%)	Largely agree (%)	Fully agree (%)
*Conduct of health care professionals*				
Psychiatric health care professionals should ask about sexuality and relationships.	1.9	12.9	13.0	72.2
Healthcare staff should routinely ask questions about sexual abuse.	1.9	11.0	20.4	66.7
It is important to meet the same health care professional during repeated visits to a health care clinic.	0	13.0	57.4	29.6
*Information and education*				
Information about how sexuality and relationships may be experienced by people with ADHD should emphasize that each individual has unique needs and resources.	0	5.6	7.4	87.0
Health care professionals should provide information about websites that address how sexuality and relationships may be experienced by people with ADHD.	0	9.3	37.0	53.7
There should be access to discussion groups for young women, regardless of their diagnosis, that address sexuality and relationships.	1.9	16.6	31.5	50.0
*Contraception counselling*				
Health care professionals should talk with me about my choice of contraceptive.	0	9.4	13.2	77.4
During contraceptive counselling, it may help to know how long the conversation will last.	1.9	12.9	9.3	75.9

The remaining eight statements in round 2 that did not reach consensus are presented in Table 5. Finally, results from both rounds were reported separately for women and HCPs in Tables S6 and S7. In most statements, the two groups were generally in agreement, including those that did not reach consensus in the group as a whole. However, statements on SRH discussion groups for young women with ADHD and for women regardless of diagnosis reached consensus only among the women, although 70% and 76% of HCPs, respectively, leaned towards the positive side. For the statement on whether HCPs should be aware of a woman's ADHD diagnosis during contraceptive counselling, neither group reached consensus.

**Table 5:** Statements not reaching consensus in round 2. *N* = 54, young women with attention deficit/hyperactivity disorder (ADHD) and health care professionals.

	Do not agree at all (%)	Partially agree, %	Largely agree, %	Fully agree, %
*Conduct of health care professionals*				
During conversations about sexuality or relationships, health care professionals should ask if I want to have someone important to me to be present for support.	11.1	44.5	18.5	25.9
It is important to be able to choose whether I want to meet the same health care staff during repeated visits to a healthcare clinic.	0	18.5	38.9	42.6
*Information and education*				
There should be access to discussion groups targeted at young women with ADHD that address sexuality and relationships.	3.7	13.0	42.6	40.7
There should be an opportunity to meet with a midwife for contraceptive counselling in connection with psychiatric visits.	15.1	28.3	28.3	28.3
*Accessibility and availability of health care*				
It is important to be able to choose the time and day for visits to a health care clinic.	1.9	38.5	36.5	23.1
*Contraceptive counselling*				
It is important that health care professionals are aware that I have an ADHD diagnosis during contraceptive counselling.	5.6	16.7	37.0	40.7
If I choose to abstain from using contraceptives, health care professionals should discuss my decision with me.	5.6	25.9	31.5	37.0
During contraceptive counselling, it may help to have the option to divide the counselling into two or more shorter sessions.	14.8	29.7	18.5	37.0

## 4 Discussion

This study identified a variety of important components in health care settings that could promote SRH among young women with ADHD. The results were based on consensus from a heterogeneous expert group, including young women with ADHD and HCPs working within youth health, school health, adult sexual health and psychiatric care. A majority of the statements tested reached consensus, with a broad agreement between women and HCPs.

The high number of statements reaching consensus is a central finding, which may suggest the importance of offering support across multiple areas of SRH and in diverse clinical settings. Given the breadth and complexity of SRH, and the variation in functional abilities among young women with ADHD, a variety of support options can be assumed to be needed [1, 52]. At the same time, the findings should also be interpreted with the understanding that a high level of consensus may not only reflect shared perspectives, but may also partly reflect what participants perceive as good clinical practice or a social desirability to endorse broadly valued principles, such as providing non‐judgemental care [48]. Including women with lived experience may have helped to reduce this influence, as their perspectives are less tied to professional norms and help situate the findings in everyday needs. Despite potential differences, the heterogeneous expert group, comprising HCPs from different clinics and women with ADHD, still largely agreed, which lends weight to the results and underscores the value of designing diverse and flexible support interventions [42].

An important finding in this study was the consensus (> 80% fully agreed) among experts regarding the need for HCPs working in psychiatric settings or SRH services to receive training that provides general knowledge about ADHD, including how sexuality and relationships may be experienced by young women with ADHD. The high level of agreement on education suggests that this competence is considered important but may also indicate a current lack of knowledge. This interpretation is consistent with previous research, where midwives expressed a need for increased knowledge about specific challenges and symptoms of ADHD among young women to improve the standard of care in youth clinics [36]. Sweden's national care and interventions programme similarly emphasizes the need for competence development among HCPs working in primary care and school health services [29]. Such knowledge may be particularly relevant in SRH contexts, where understanding how ADHD affects everyday functioning may reduce the risk of patients feeling judged or encountering prejudice in healthcare encounters, a risk previously reported among individuals with ADHD [33, 45]. The need for knowledge may also be reflected in the lack of consensus in our findings on whether HCPs should be aware of a woman's ADHD diagnosis during contraceptive counselling. This might relate to concerns about stigma, fear of judgement or reluctance to disclose ADHD in SRH contexts, although this interpretation requires further exploration. Even though HCPs working in psychiatric settings can be assumed to have a general understanding of ADHD, providing training to help them address questions about sexuality and relationships may still be important, especially as addressing sexuality in psychiatric settings was considered an important component of our study. Research suggests that these topics may not be raised, often due to discomfort or concerns about causing patients to feel shame [53]. Taken together, knowledge of ADHD may support person‐centred care by helping HCPs in psychiatric settings and SRH services to better understand and respond to each individual's unique needs, preferences and challenges [32].

The need for a broader understanding that is not confined to a single clinical area was further underscored by the high level of agreement in our results regarding the menstrual cycle. In Sweden, problems related to PMS are typically treated in youth clinics or gynaecological clinics, but the expert group also emphasized that HCPs in psychiatric settings, prescribing and monitoring ADHD medication, should be aware of fluctuations of symptoms during the menstrual cycle. The high agreement in the expert group may reflect previous research demonstrating that hormonal changes can exacerbate ADHD symptoms and that women with ADHD are at increased risk of severe PMS [54, 55]. Greater knowledge of these factors may make HCPs more likely to consider recommended individual treatment adjustments in ways that support both symptom management and overall well‐being.

This study further underscores several important components of health care, namely provision of accessible information, education and SRH counselling for young women with ADHD to promote SRH. These components all align with WHO's guidelines for youth‐friendly health services [56]. However, our findings highlight strategies that may be especially important for young women with ADHD and how they could be adapted. The expert group agreed that written information should be available, both at health clinics and online, that addresses how sexuality and relationships can be experienced by people with ADHD. However, information tailored toward people with ADHD must be developed with caution to not reinforce stigma. According to the expert group, information needed to emphasize that individuals have unique needs and resources, which could be important to avoid generalization and stereotyping [57]. Highlighting individual variation may also be important given the variety of symptoms of ADHD and functional abilities among young women [7]. Ashton et al. [58] further emphasized the value of involving people with lived experience in the development of educational content to avoid stigmatization. To make information and SRH counselling available and accessible, this study further highlights the importance of having a structured approach to contraceptive counselling in health encounters and offering flexible appointment formats and opening hours in youth clinics. These strategies could probably benefit all young adults, regardless of diagnosis [59]. However, considering the association between ADHD symptoms and difficulties with attention and time management, such approaches may be particularly valuable to young women with ADHD [7]. The expert group further agreed on the importance of discussion groups for young women in general. Online peer‐group interventions in young people with mental health problems have shown positive outcomes on improving self‐knowledge and reducing self‐stigma [60], strengthen our findings. However, discussion groups directed at young women with ADHD did not reach consensus in our findings, although more than 80% of the experts leaned towards a positive appraisal. This may relate to limited time and staffing resources in both sexual health and psychiatric services, a pattern described in previous research where HCPs expressed willingness to improve care but reported insufficient organizational support [36, 61]. To manage limited resources, HCPs in an earlier study suggested combining women with different diagnoses, such as autism spectrum disorder or personality disorders, into shared discussion groups concerning SRH [40]. However, it is important to note that when the data were separated for women and HCPs, only the women reached consensus regarding discussion groups for young women in general and for groups directed at women with ADHD. This may indicate that discussion groups are viewed as important by the women and should therefore be considered a priority in health care. The expert group in the present study also supported incorporating sexuality and relationships topics into existing psychoeducational groups within psychiatric care, aligning with a review showing that such content is often absent from current psychoeducational interventions for adults [62]. Structured group‐based psychoeducation has been shown to increase knowledge and life satisfaction, and including SRH‐topics in an existing education may help facilitate meaningful discussions, particularly in settings where clinical resources are constrained [63].

## 5 Strengths and Limitations

To our knowledge, this is the first Delphi study aimed at identifying important components for promoting SRH among young women with ADHD. In Delphi studies, the diversity and expertise of the panel are often more critical than the sample size [64]. This study benefited from having a heterogeneous expert group. The diversity of the expert panel enriched the understanding of how various services can support SRH, thereby not only enhancing the credibility of the proposed components but also increasing their potential for practical implementation across different settings [42].

Although the high number of statements reaching consensus suggests shared needs among young women with ADHD, it is important to acknowledge that not all identified components can be considered as universally true or applicable to all women [37]. The variation in functional abilities and symptom profiles among these women must also be considered [7]. Additionally, the results may be influenced by selection bias, as women with greater support needs or HCPs interested in the topic might have been more motivated to participate [65]. ADHD was also self‐reported and could not be verified, limiting representation and generalization of data. Transferability of the results further needs to take context into account, including possible differences in health care structures between countries and the cultural and social factors that influence SRH. Moreover, ethnicity of the participating women was not collected, which may limit understanding of how the findings apply across diverse groups [1, 66].

Furthermore, additional Delphi rounds might have led to more statements reaching consensus, but extending the process beyond two rounds could have risked participant fatigue and dropout, potentially reducing the diversity of perspectives [43]. Given previous challenges in recruiting young women with ADHD in this research group, the decision to limit the study to two rounds was deliberate. However, the use of an online survey platform, combined with multiple electronic reminders, appeared well‐suited to the target group. This approach resulted in relatively little attrition in the second round, supporting the study's credibility and ensuring participant anonymity [67]. The dropouts included both women (*n* = 4) and HCPs (*n* = 5), resulting in a roughly balanced loss across groups. However, no women aged 15–18 years participated, limiting the results. As previous studies have shown that recruiting adolescents to research can be challenging [68] and SRH may be perceived as a sensitive topic, future studies targeting this age group may be beneficial to capture their perspectives.

## 6 Conclusion and Future Directions

The study identified key components within health care settings that may support SRH among young women with ADHD. Young women may benefit from comprehensive, person‐centred support delivered across both sexual health and psychiatric services. Providing accessible care through structured clinical encounters, flexible appointment options, and routine attention to sexuality, relationships, and the menstrual cycle within clinical practice and psychoeducation could contribute to more supportive care environments. The study also highlighted the importance of non‐judgmental encounters and the need for training in SRH and ADHD among health care personnel. Further research should evaluate how these components can be effectively implemented in clinical practice and assess their impact on SRH outcomes for young women with ADHD.

## Author Contributions

**Karin Wallin:** conceptualization, methodology, formal analysis, writing – original draft (lead), writing – review and editing (equal). **Sally Hultsjö, Inger Wallin‐Lundell, Siw Alehagen** and **Lena Hanberger:** conceptualization and methodology (supporting), review and editing (equal). All authors have contributed to the manuscript and approved the final version.

## Ethics Statement

This research was conducted ethically in accordance with the World Medical Association Declaration of Helsinki and approved by the Swedish Research Ethics Committee (approval no.2023‐03566‐01) on September 8th, 2023.

## Consent

All participants provided written informed consent before enrolment in the study. According to Swedish legislation, minors over the age of 15 are allowed to consent without the consent of a legal guardian.

## Conflicts of Interest

The authors declare no conflicts of interest.

## Supporting information


Supporting File

## Data Availability

The datasets generated during and/or analyzed during the current study are available only on request from the corresponding author due to ethical restrictions.

## References

[1] A. M. Starrs , A. C. Ezeh , G. Barker , et al., “Accelerate Progress—Sexual and Reproductive Health and Rights for All: Report of the Guttmacher–Lancet Commission,” Lancet 391, no. 10140 (2018): 2642–2692. 29753597 10.1016/S0140-6736(18)30293-9

[2] World Health Organization . (2006). Defining Sexual Health‐Report of a Technical Consultation on Sexual Health 28–31 January 2002. Geneva.

[3] C. T. Halpern and C. E. Kaestle , “Sexuality in Emerging Adulthood,” in APA Handbook of Sexuality and Psychology, Vol. 1: Person‐Based Approaches (American Psychological Association, 2014), 487–522.

[4] D. Van de Bongardt , R. Yu , M. Deković , and W. H. Meeus , “Romantic Relationships and Sexuality in Adolescence and Young Adulthood: The Role of Parents, Peers, and Partners,” in *Romantic Relationships and Sexuality in Adolescence and Young Adulthood* (Routledge, 2018), 13–31.

[5] A. G. Eng , U. Nirjar , A. R. Elkins , et al., “Attention‐Deficit/Hyperactivity Disorder and the Menstrual Cycle: Theory and Evidence,” Hormones and Behavior 158 (2024): 105466, 10.1016/j.yhbeh.2023.105466. 38039899 PMC10872410

[6] M. O'Brien , C. Kini‐Seery , C. Kelly , et al., “‘I Felt Like a Burden’: An Exploration Into the Experience of Romantic Relationships for People With ADHD,” Journal of Marital and Family Therapy 52, no. 1 (2026): e70097. 41315883 10.1111/jmft.70097 PMC12662942

[7] American Psychiatric Association , *Diagnostic and Statistical Manual of Mental Disorders: DSM‐5* (American Psychiatric Association, 2013) (5th ed.).

[8] K. Wallin , I. Wallin Lundell , L. Hanberger , S. Alehagen , and S. Hultsjö , “Self‐Experienced Sexual and Reproductive Health in Young Women With Attention Deficit Hyperactivity Disorder a Qualitative Interview Study,” BMC Women's Health 22, no. 1 (2022): 289, 10.1186/s12905-022-01867-y. 35836208 PMC9281117

[9] S. Young , L. J. Klassen , S. D. Reitmeier , J. D. Matheson , and G. H. Gudjonsson , “Let's Talk about Sex and ADHD: Findings From an Anonymous Online Survey,” International Journal of Environmental Research and Public Health 20, no. 3 (2023): 2037, https://mdpi-res.com/d_attachment/ijerph/ijerph-20-02037/article_deploy/ijerph-20-02037-v2.pdf?version=1675218891. 36767401 10.3390/ijerph20032037 PMC9915044

[10] P. G. Hertz , D. Turner , S. Barra , et al., “Sexuality in Adults With ADHD: Results of an Online Survey,” Frontiers in Psychiatry 13 (2022): 868278, 10.3389/fpsyt.2022.868278. 35651826 PMC9148957

[11] T. Jensen‐Fogt and C. L. Pedersen , “Exploring Attention‐Deficit/Hyperactivity Disorder (ADHD) Symptomatology in Relation to Women's Orgasmic Consistency,” Journal of Sex Research 21 (2025): 1–11, 10.1080/00224499.2025.2489771. 40257106

[12] M. R. Bruner , A. D. Kuryluk , and S. W. Whitton , “Attention‐Deficit/Hyperactivity Disorder Symptom Levels and Romantic Relationship Quality in College Students,” Journal of American College Health 63, no. 2 (2015): 98–108, 10.1080/07448481.2014.975717. 25350392

[13] G. M. M. Hosain , A. B. Berenson , H. Tennen , L. O. Bauer , and Z. H. Wu , “Attention Deficit Hyperactivity Symptoms and Risky Sexual Behavior in Young Adult Women,” Journal of Women's Health 21, no. 4 (2012): 463–468, 10.1089/jwh.2011.2825. PMC3321673 22303821

[14] S. P. Huggins , M. E. Rooney , and A. Chronis‐Tuscano , “Risky Sexual Behavior Among College Students With ADHD: Is the Mother‐Child Relationship Protective?,” Journal of Attention Disorders 19, no. 3 (2015): 240–250, 10.1177/1087054712459560. 23048048

[15] J. Isaksson , A. Stickley , R. Koposov , and V. Ruchkin , “The Danger of Being Inattentive ‐ ADHD Symptoms and Risky Sexual Behaviour in Russian Adolescents,” European Psychiatry 47 (2018): 42–48, 10.1016/j.eurpsy.2017.09.004. 29100171

[16] J. A. Snyder , “The Link Between ADHD and the Risk of Sexual Victimization Among College Women: Expanding the Lifestyles/Routine Activities Framework,” Violence Against Women 21, no. 11 (2015): 1364–1384, 10.1177/1077801215593647. 26155795

[17] C. Skoglund , H. Kopp Kallner , A. Skalkidou , et al., “Association of Attention‐Deficit/Hyperactivity Disorder With Teenage Birth Among Women and Girls in Sweden,” JAMA Network Open 2, no. 10 (2019): e1912463, 10.1001/jamanetworkopen.2019.12463. 31577361 PMC6777395

[18] M. H. Chen , J. W. Hsu , K. L. Huang , et al., “Sexually Transmitted Infection Among Adolescents and Young Adults With Attention‐Deficit/Hyperactivity Disorder: A Nationwide Longitudinal Study,” Journal of the American Academy of Child & Adolescent Psychiatry 57, no. 1 (2018): 48–53, 10.1016/j.jaac.2017.09.438. 29301669

[19] S. Young , N. Adamo , B. B. Ásgeirsdóttir , et al., “Females With ADHD: An Expert Consensus Statement Taking a Lifespan Approach Providing Guidance for the Identification and Treatment of Attention‐Deficit/Hyperactivity Disorder in Girls and Women,” BMC Psychiatry 20, no. 1 (2020): 404, 10.1186/s12888-020-02707-9. 32787804 PMC7422602

[20] E. Holden and H. Kobayashi‐Wood , “Adverse Experiences of Women With Undiagnosed ADHD and the Invaluable Role of Diagnosis,” Scientific Reports 15, no. 1 (2025): 20945, 10.1038/s41598-025-04782-y. 40594310 PMC12218314

[21] P. O. Quinn and M. Madhoo , “A Review of Attention‐Deficit/Hyperactivity Disorder in Women and Girls: Uncovering This Hidden Diagnosis,” Primary Care Companion CNS Disorders 16, no. 3 (2014): 10.4088/PCC.13r01596. PMC4195638 25317366

[22] V. Ozdag , S. Yuce , and N. Soylu , “Online Sexual Abuse, Sexting, and Bullying Among Adolescents With and Without ADHD: A Cross‐Sectional Study in a Turkish Sample,” Acta Psychologica 260 (2025): 105688. 41066854 10.1016/j.actpsy.2025.105688

[23] Y. Pollak , T. J. Dekkers , R. Shoham , and H. M. Huizenga , “Risk‐Taking Behavior in Attention Deficit/Hyperactivity Disorder (ADHD): A Review of Potential Underlying Mechanisms and of Interventions,” Current Psychiatry Reports 21, no. 5 (2019): 33. 30903380 10.1007/s11920-019-1019-y

[24] R. Alarcon‐Rodriguez , R. García‐Álvarez , R. Fadul‐Calderon , et al., “The Relationship Between Female Orgasmic Disorder, Attention‐Deficit/Hyperactivity Disorder, and Depression in Dominican Women,” Journal of Sexual Medicine 21, no. 7 (2024): 614–619, 10.1093/jsxmed/qdae048. 38628064

[25] D. Dèttore , M. Pucciarelli , and E. Santarnecchi , “Anxiety and Female Sexual Functioning: An Empirical Study,” Journal of Sex & Marital Therapy 39, no. 3 (2013): 216–240. 23356511 10.1080/0092623X.2011.606879

[26] E. Fuller‐Thomson , D. A. Lewis , and S. K. Agbeyaka , “Attention‐Deficit/Hyperactivity Disorder Casts a Long Shadow: Findings From a Population‐Based Study of Adult Women With Self‐Reported ADHD,” Child: Care, Health and Development 42, no. 6 (2016): 918–927, 10.1111/cch.12380. 27439337

[27] Association of Youth Clinics in Sweden . (2024). Handbook for Youth Clinics in Sweden. Retrieved 251010 From Handbok-24.pdf.

[28] Public Health Agency of Sweden . (2020). National Strategy for Sexual and Reproductive Health and Rights (SRHR).

[29] Swedish Association of Local Authorities and Regions . (2023). National Care and Intervention Program for ADHD. Received 251005 From https://www.vardochinsats.se/adhd/.

[30] Youth Clinic Online. umo.se‐om sex, hälsa och relationer . [umo.se‐about sex, health and relationships]. (2026) [cited July 15]. https://www.umo.se/.

[31] Swedish National Health Care Guidance 1177.se . (2026) [cited July 15]. https://www.1177.se.

[32] I. Ekman , K. Swedberg , C. Taft , et al., “Person‐Centered Care—Ready for Prime Time,” European Journal of Cardiovascular Nursing 10, no. 4 (2011): 248–251, 10.1016/j.ejcnurse.2011.06.008. 21764386

[33] T. V. Masuch , M. Bea , B. Alm , P. Deibler , and E. Sobanski , “Internalized Stigma, Anticipated Discrimination and Perceived Public Stigma in Adults With ADHD,” ADHD Attention Deficit and Hyperactivity Disorders 11, no. 2 (2019): 211–220, 10.1007/s12402-018-0274-9. 30341693

[34] M. S. Argenyi and T. G. James , “Sexual Risk Behavior and Sexually Transmitted Infections Among College Students With Disabilities,” Sexually Transmitted Diseases 48, no. 11 (2021): 851–854, 10.1097/olq.0000000000001443. 33872223

[35] M. Smusz , C. Birkbeck , A. Bidgood , and C. S. Allely , “Exploring the Experience of Romantic Relationships and Sexuality Education in Neurodivergent and Neurotypical Young Individuals,” Sexuality and Disability 42 (2024): 735–764.

[36] A. K. Klint Carlander , M. Thorsell , Y. Demetry , S. Nikodell , H. Kopp Kallner , and C. Skoglund , “Knowledge, Challenges, and Standard of Care of Young Women With ADHD at Swedish Youth Clinics,” Sexual & Reproductive Healthcare 32 (2022): 100727, 10.1016/j.srhc.2022.100727. 35461165

[37] S. Keeney , F. Hasson , and H. McKenna , “Consulting the Oracle: Ten Lessons From Using the Delphi Technique in Nursing Research,” Journal of Advanced Nursing 53, no. 2 (2006): 205–212. https://onlinelibrary.wiley.com/doi/pdfdirect/10.1111/j.1365-2648.2006.03716.x?download=true. 16422719 10.1111/j.1365-2648.2006.03716.x

[38] United Nations , *Transforming Our World: The 2030 Agenda for Sustainable Development* (Department of Economic and Social Affairs, United Nations, 2015).

[39] J. Petkovic , A. Riddle , E. A. Akl , et al., “Protocol for the Development of Guidance for Stakeholder Engagement in Health and Healthcare Guideline Development and Implementation,” Systematic Reviews 9, no. 1 (2020): 21, 10.1186/s13643-020-1272-5. 32007104 PMC6995157

[40] K. Wallin , I. Wallin‐Lundell , S. Alehagen , L. Hanberger , and S. Hultsjö , “Having Reliable Support: A Prerequisite to Promote Sexual and Reproductive Health in Young Women With ADHD,” Archives of Sexual Behavior 53 (2024): 4117–4129, 10.1007/s10508-024-03001-5. 39313694 PMC11588869

[41] H. A. Linstone and M. Turoff , *The Delphi Method: Techniques and Applications* (Addison‐Wesley, 1975).

[42] R. Boulkedid , H. Abdoul , M. Loustau , O. Sibony , and C. Alberti , “Using and Reporting the Delphi Method for Selecting Healthcare Quality Indicators: A Systematic Review,” PLoS One 6, no. 6 (2011): e20476, 10.1371/journal.pone.0020476. 21694759 PMC3111406

[43] H. P. McKenna , “The Delphi Technique: A Worthwhile Research Approach for Nursing?,” Journal of Advanced Nursing 19, no. 6 (1994): 1221–1225, 10.1111/j.1365-2648.1994.tb01207.x. 7930104

[44] M. Niederberger and J. Spranger , “Delphi Technique in Health Sciences: A Map,” Frontiers in Public Health 8 (2020): 457. 33072683 10.3389/fpubh.2020.00457 PMC7536299

[45] K. Wallin , S. Alehagen , L. Hanberger , I. W. Lundell , and S. Hultsjö , “Sexual and Reproductive Health in Young Women With ADHD From the View of Health Care Professionals,” BMC Women's Health 24, no. 1 (2024): 389, 10.1186/s12905-024-03230-9. 38970031 PMC11225155

[46] B. Roberts , T. Eisenlohr‐Moul , and M. M. Martel , “Reproductive Steroids and ADHD Symptoms Across the Menstrual Cycle,” Psychoneuroendocrinology 88 (2018): 105–114, 10.1016/j.psyneuen.2017.11.015. 29197795 PMC5803442

[47] M. de Jong , D. S. M. R. Wynchank , E. van Andel , A. T. F. Beekman , and J. J. S. Kooij , “Female‐Specific Pharmacotherapy in ADHD: Premenstrual Adjustment of Psychostimulant Dosage,” Frontiers in Psychiatry 14 (2023): 1306194, 10.3389/fpsyt.2023.1306194. 38152361 PMC10751335

[48] J. Schifano and M. Niederberger , “How Delphi Studies in the Health Sciences Find Consensus: A Scoping Review,” Systematic Reviews 14, no. 1 (2025): 14, 10.1186/s13643-024-02738-3. 39810238 PMC11734368

[49] The Swedish Act . (2003: 460) om etikprövning av forskning som avser människor [The Act Concerning the Ethical Review of Research Involving Humans].

[50] A. M. Manyara , A. Purvis , O. Ciani , G. S. Collins , and R. S. Taylor , “Sample Size in Multistakeholder Delphi Surveys: At What Minimum Sample Size Do Replicability of Results Stabilize?,” Journal of Clinical Epidemiology 174 (2024): 111485, 10.1016/j.jclinepi.2024.111485. 39069013 PMC7617918

[51] S. Hultsjö , C. Berterö , H. Arvidsson , and K. Hjelm , “Core Components in the Care of Immigrants With Psychoses: A Delphi Survey of Patients, Families, and Health‐Care Staff,” International Journal of Mental Health Nursing 20, no. 3 (2011): 174–184, 10.1111/j.1447-0349.2010.00720.x. 21352448

[52] S. Young , D. Moss , O. Sedgwick , M. Fridman , and P. Hodgkins , “A Meta‐Analysis of the Prevalence of Attention Deficit Hyperactivity Disorder in Incarcerated Populations,” Psychological Medicine 45, no. 2 (2015): 247–258, 10.1017/s0033291714000762. 25066071 PMC4301200

[53] S. L. Bungener , L. Post , I. Berends , T. D. Steensma , A. L. C. de Vries , and A. Popma , “Talking About Sexuality With Youth: A Taboo in Psychiatry?,” Journal of Sexual Medicine 19, no. 3 (2022): 421–429, 10.1016/j.jsxm.2022.01.001. 35105513

[54] F. Dorani , D. Bijlenga , A. T. F. Beekman , E. J. W. van Someren , and J. J. S. Kooij , “Prevalence of Hormone‐Related Mood Disorder Symptoms in Women With ADHD,” Journal of Psychiatric Research 133 (2021): 10–15, 10.1016/j.jpsychires.2020.12.005. 33302160

[55] J. J. S. Kooij , M. de Jong , J. Agnew‐Blais , et al., “Research Advances and Future Directions in Female ADHD: The Lifelong Interplay of Hormonal Fluctuations With Mood, Cognition, and Disease,” Frontiers in Global Women's Health 6 (2025): 2025, 10.3389/fgwh.2025.1613628. PMC12277363 40692967

[56] World Health Organization , *Global Consultation on Adolescent Friendly Health Services‐A Consensus Statement* (World Health Organization, 2002), 29.

[57] A. Kågström , Z. Guerrero , A. A. Aliev , et al., “Mental Health Stigma and Its Consequences: A Systematic Scoping Review of Pathways to Discrimination and Adverse Outcomes,” eClinicalMedicine 89 (2025): 103588, 10.1016/j.eclinm.2025.103588. 41210382 PMC12593596

[58] L. J. Ashton , S. E. Gordon , and R. A. Reeves , “Key Ingredients‐Target Groups, Methods and Messages, and Evaluation‐of Local‐Level, Public Interventions to Counter Stigma and Discrimination: A Lived Experience Informed Selective Narrative Literature Review,” Community Mental Health Journal 54, no. 3 (2018): 312–333, 10.1007/s10597-017-0189-5. 29185150

[59] S. Thomée , D. Malm , M. Christianson , et al., “Challenges and Strategies for Sustaining Youth‐Friendly Health Services—A Qualitative Study From the Perspective of Professionals at Youth Clinics in Northern Sweden,” Reproductive Health 13, no. 1 (2016): 147, 10.1186/s12978-016-0261-6. 28003025 PMC5178097

[60] S. Yuan , G. Davidson , and P. Best , “Online Mental Health Peer Support: A Systematic Scoping Review of Theoretical Mechanisms of Effect,” BMC Digital Health 3, no. 1 (2025): 68.

[61] N. Eder , K. Nordenberg , N. Långström , A. Rozental , and A. Moell , “Moral Distress Among Inpatient Child and Adolescent Psychiatry Staff: A Mixed‐Methods Study of Experiences and Associated Factors,” Child and Adolescent Psychiatry and Mental Health 19, no. 1 (2025): 16, 10.1186/s13034-025-00868-7. 40022125 PMC11871634

[62] H. Pedersen , T. Skliarova , S. A. Pedersen , R. W. Gråwe , A. Havnen , and M. L. Lara‐Cabrera , “Psychoeducation for Adult ADHD: A Scoping Review About Characteristics, Patient Involvement, and Content,” BMC Psychiatry 24, no. 1 (2024): 73. 38273266 10.1186/s12888-024-05530-8 PMC10811906

[63] T. Hirvikoski , T. Lindström , J. Carlsson , E. Waaler , J. Jokinen , and S. Bölte , “Psychoeducational Groups for Adults With ADHD and Their Significant Others (PEGASUS): A Pragmatic Multicenter and Randomized Controlled Trial,” European Psychiatry 44 (2017): 141–152, 10.1016/j.eurpsy.2017.04.005. 28641216

[64] C. Okoli and S. D. Pawlowski , “The Delphi Method as a Research Tool: An Example, Design Considerations and Applications,” Information & Management 42, no. 1 (2004): 15–29, 10.1016/j.im.2003.11.002.

[65] G. Ejlertsson , Enkäten i praktiken: en handbok i enkätmetodik (Studentlitteratur, 2019).

[66] M. Q. Patton , *Qualitative Research & Evaluation Methods: Integrating Theory and Practice* (SAGE Publications, 2015), 4. ed.

[67] F. Hasson and S. Keeney , “Enhancing Rigour in the Delphi Technique Research,” Technological Forecasting and Social Change 78, no. 9 (2011): 1695–1704, 10.1016/j.techfore.2011.04.005.

[68] D. E. Parrish , J. F. Duron , and H. K. Oxhandler , “Adolescent Recruitment Strategies: Lessons Learned From a University‐Based Study of Social Anxiety,” Social Work Research 41, no. 4 (2017): 213–220, 10.1093/swr/svx016.

