# Supplementary material for: Important Health Care Components to Promote Sexual and Reproductive Health Among Young Women With Attention‐Deficit/Hyperactivity Disorder: A Delphi Study
Source: Health Expect. 2026 Sep 2;29(5):e70856. doi: 10.1111/hex.70856 (PMC13538955; doi:10.1111/hex.70856)
Supplement: Supplementary file 1 [file HEX-29-e70856-s001.docx]

**Results from round 1 and 2. Health Care Professionals and Women**

Table 6. Round 1 result by respondent category: Health care professionals (HCPs) and Women.

HCPs. n = 36, Women. n = 27

| **Statement** | **Respondent category** | **Do not agree at all** | **Partly agree** | **Largely agree** | **Fully agree** |
| --- | --- | --- | --- | --- | --- |
| It is important that I am treated without prejudice and do not feel judged in meetings with health care professionals. | HCPs | 0.0 % | 8.3 % | 5.6 % | 86.1 % |
|  | Women | 0.0 % | 0.0 % | 0.0 % | 100.0 % |
| It is important to meet the same health care professional during repeated visits to a health care facility. | HCPs | 8.3 % | 11.1 % | 44.4 % | 36.1 % |
|  | Women | 0.0 % | 7.4 % | 44.4 % | 48.1 % |
| It is important that health care professionals not only discuss the risks of sex but also highlight the positive aspects. | HCPs | 2.8 % | 8.3 % | 16.7 % | 72.2 % |
|  | Women | 0.0 % | 7.4 % | 40.7 % | 51.9 % |
| Health care professionals should routinely ask questions about sexual abuse. | HCPs | 2.8 % | 13.9 % | 22.2 % | 61.1 % |
|  | Women | 0.0 % | 33.3 % | 18.5 % | 48.1 % |
| During discussions about sexuality or relationships. health care professionals should ask if I want to have a significant person with me for support. | HCPs | 8.3 % | 33.3 % | 16.7 % | 41.7 % |
|  | Women | 14.8 % | 40.7 % | 11.1 % | 33.3 % |
| Health care professionals in psychiatry should ask about sexuality and relationships. | HCPs | 0.0 % | 11.1 % | 22.2 % | 66.7 % |
|  | Women | 3.7 % | 33.3 % | 33.3 % | 29.6 % |
| Health care professionals should inform about websites that address how sexuality and relationships can be experienced by people with ADHD. | HCPs | 2.9 % | 14.7 % | 50.0 % | 32.4 % |
|  | Women | 3.7 % | 3.7 % | 22.2 % | 70.4 % |
| There should be written information on 1177.se describing how sexuality and relationships can be experienced by people with ADHD. | HCPs | 0.0 % | 2.9 % | 23.5 % | 73.5 % |
|  | Women | 3.7 % | 3.7 % | 14.8 % | 77.8 % |
| There should be video links on 1177.se that address how sexuality and relationships can be experienced by people with ADHD. | HCPs | 2.9 % | 5.9 % | 29.4 % | 61.8 % |
|  | Women | 7.4 % | 14.8 % | 22.2 % | 55.6 % |
| There should be written information at sexual health clinics* describing how sexuality and relationships can be experienced by people with ADHD (*youth clinics. school health. gynecological clinics. midwifery clinics. RFSU clinics). | HCPs | 0.0 % | 3.0 % | 27.3 % | 69.7 % |
|  | Women | 0.0 % | 7.4 % | 22.2 % | 70.4 % |
| Sexuality and relationships should be included in training provided within psychiatry to people with ADHD. | HCPs | 0.0 % | 5.7 % | 14.3 % | 80.0 % |
|  | Women | 0.0 % | 11.1 % | 22.2 % | 66.7 % |
| Healthcare professionals in psychiatry should receive training on how women with ADHD can experience sexuality and relationships. | HCPs | 0.0 % | 5.7 % | 11.4 % | 82.9 % |
|  | Women | 0.0 % | 7.4 % | 0.0 % | 92.6 % |
| There should be access to discussion groups aimed at women with ADHD that address sexuality and relationships. | HCPs | 5.7 % | 14.3 % | 45.7 % | 34.3 % |
|  | Women | 3.7 % | 7.4 % | 29.6 % | 59.3 % |
| Health care professionals at sexual health clinics* should receive training about ADHD (*youth clinics. school health. gynecological clinics. midwifery clinics. RFSU clinics). | HCPs | 0.0 % | 2.9 % | 14.7 % | 82.4 % |
|  | Women | 0.0 % | 7.4 % | 3.7 % | 88.9 % |
| There should be drop-in times at youth clinics. | HCPs | 2.9 % | 5.7 % | 25.7 % | 65.7 % |
|  | Women | 0.0 % | 3.7 % | 7.4 % | 88.9 % |
| Youth clinics should have evening hours. | HCPs | 0.0 % | 11.4 % | 42.9 % | 45.7 % |
|  | Women | 0.0 % | 22.2 % | 18.5 % | 59.3 % |
| There should be the possibility of digital meetings at youth clinics. | HCPs | 0.0 % | 2.9 % | 20.0 % | 77.1 % |
|  | Women | 0.0 % | 14.8 % | 37.0 % | 48.1 % |
| It is important that health care professionals help me contact other clinics if they cannot provide the support I need. | HCPs | 0.0 % | 5.7 % | 20.0 % | 74.3 % |
|  | Women | 0.0 % | 3.7 % | 7.4 % | 88.9 % |
| It should be possible to contact health care services anonymously for advice on sexuality and relationships. | HCPs | 5.6 % | 5.6 % | 33.3 % | 55.6 % |
|  | Women | 0.0 % | 3.7 % | 37.0 % | 59.3 % |
| Health care professionals should not question my choice of contraception. | HCPs | 0.0 % | 25.7 % | 37.1 % | 37.1 % |
|  | Women | 11.1 % | 44.4 % | 18.5 % | 25.9 % |
| There should be an opportunity to meet a midwife for contraceptive counseling in connection with visits to psychiatry. | HCPs | 17.1 % | 22.9 % | 37.1 % | 22.9 % |
|  | Women | 3.7 % | 29.6 % | 18.5 % | 48.1 % |
| It is important that health care professionals know that I have an ADHD diagnosis during contraceptive counseling. | HCPs | 11.8 % | 11.8 % | 41.2 % | 35.3 % |
|  | Women | 7.4 % | 18.5 % | 33.3 % | 40.7 % |
| Health care professionals should not question if I choose to abstain from contraception. | HCPs | 2.9 % | 31.4 % | 37.1 % | 28.6 % |
|  | Women | 11.1 % | 18.5 % | 33.3 % | 37.0 % |
| During contraceptive counseling it. may help to know how long the conversation will take. | HCPs | 0.0 % | 8.6 % | 25.7 % | 65.7 % |
|  | Women | 7.4 % | 25.9 % | 14.8 % | 51.9 % |
| During contraceptive counseling. it can be helpful to know what the conversation will include. | HCPs | 0.0 % | 2.9 % | 22.9 % | 74.3 % |
|  | Women | 7.4 % | 18.5 % | 25.9 % | 48.1 % |
| During contraceptive counseling. it can be helpful to repeat verbal information. | HCPs | 0.0 % | 0.0 % | 25.7 % | 74.3 % |
|  | Women | 7.4 % | 14.8 % | 29.6 % | 48.1 % |
| During contraceptive counseling. it can be helpful to receive written information. | HCPs | 0.0 % | 2.9 % | 25.7 % | 71.4 % |
|  | Women | 3.7 % | 0.0 % | 14.8 % | 81.5 % |
| During contraceptive counseling. it can be helpful to have the opportunity to divide the counseling into two or more shorter meetings. | HCPs | 0.0 % | 14.3 % | 25.7 % | 60.0 % |
|  | Women | 33.3 % | 29.6 % | 3.7 % | 33.3 % |
| During contraceptive counseling. it can be helpful to receive a summary of what was said in the conversation. | HCPs | 0.0 % | 5.7 % | 25.7 % | 68.6 % |
|  | Women | 3.7 % | 7.4 % | 14.8 % | 74.1 % |
| Health care professionals should be aware that women can have more symptoms of ADHD during certain parts of the menstrual cycle. | HCPs | 0.0 % | 0.0 % | 11.8 % | 88.2 % |
|  | Women | 3.7 % | 0.0 % | 3.7 % | 92.6 % |
| When ADHD medication is prescribed or followed up. health care professionals should ask how the menstrual cycle affects my ADHD symptoms. | HCPs | 0.0 % | 8.6 % | 11.4 % | 80.0 % |
|  | Women | 11.1 % | 3.7 % | 11.1 % | 74.1 % |
| Health care professionals should be aware that severe PMS (physical. emotional. and behavioral problems before menstruation) is more common in women with ADHD. | HCPs | 0.0 % | 0.0 % | 5.9 % | 94.1 % |
|  | Women | 3.7 % | 3.7 % | 11.1 % | 81.5 % |

Table 7. Round 2 result by respondent category: Health care professionals (HCPs) and Women

HCPs n = 31, Women n = 23

| **Statement** | **Respondent category** | **Do not agree at all** | **Partly agree** | **Largely agree** | **Fully agree** |
| --- | --- | --- | --- | --- | --- |
| It is important to meet the same healthcare staff during repeated visits to a healthcare clinic. | HCPs | 0.0% | 16.1% | 58.1% | 25.8% |
|  | Women | 0.0% | 8.7% | 56.5% | 34.8% |
| It is important to be able to choose whether I want to meet the same health care staff during repeated visits to a healthcare clinic. | HCPs | 0.0% | 25.8% | 38.7% | 35.5% |
|  | Women | 0.0% | 8.7% | 39.1% | 52.2% |
| It is important to be able to choose the time and day for visits to a health care clinic. | HCPs | 3.3% | 46.7% | 36.7% | 13.3% |
|  | Women | 0.0% | 27.3% | 36.4% | 36.4% |
| Health care staff should routinely ask questions about sexual abuse. | HCPs | 0.0% | 3.2% | 22.6% | 74.2% |
|  | Women | 4.3% | 21.7% | 17.4% | 56.5% |
| During conversations about sexuality or relationships. HCPs should ask if I want to have someone important to me to be present for support. | HCPs | 9.7% | 38.7% | 19.4% | 32.3% |
|  | Women | 13.0% | 52.2% | 17.4% | 17.4% |
| Psychiatric health care professionals should ask about sexuality and relationships. | HCPs | 0.0% | 0.0% | 12.9% | 87.1% |
|  | Women | 4.3% | 30.4% | 13.0% | 52.2% |
| Health care professionals should provide information about websites that address how sexuality and relationships may be experienced by people with ADHD. | HCPs | 0.0% | 12.9% | 48.4% | 38.7% |
|  | Women | 0.0% | 8.7% | 21.7% | 69.6% |
| Information about how sexuality and relationships may be experienced by people with ADHD should emphasize that each individual has unique needs and resources. | HCPs | 0.0% | 3.2% | 6.5% | 90.3% |
|  | Women | 0.0% | 8.7% | 8.7% | 82.6% |
| There should be access to discussion groups targeted at young women with ADHD that address sexuality and relationships. | HCPs | 6.5% | 16.1% | 51.6% | 25.8% |
|  | Women | 0.0% | 4.3% | 34.8% | 60.9% |
| There should be access to discussion groups for young women. regardless of their diagnosis. that address sexuality and relationships. | HCPs | 3.3% | 26.7% | 40.0% | 30.0% |
|  | Women | 0.0% | 0.0% | 21.7% | 78.3% |
| Health care professionals should talk with me about my choice of contraceptive. | HCPs | 0.0% | 6.5% | 12.9% | 80.6% |
|  | Women | 0.0% | 13.6% | 13.6% | 72.7% |
| There should be an opportunity to meet with a midwife for contraceptive counseling in connection with psychiatric visits. | HCPs | 20.0% | 30.0% | 23.3% | 26.7% |
|  | Women | 8.7% | 21.7% | 34.8% | 34.8% |
| It is important that HCPs are aware that I have an ADHD diagnosis during contraceptive counseling. | HCPs | 3.2% | 19.4% | 38.7% | 38.7% |
|  | Women | 8.7% | 13.0% | 30.4% | 47.8% |
| If I choose to abstain from using contraceptives. health care professionals should discuss my decision with me. | HCPs | 6.5% | 29.0% | 25.8% | 38.7% |
|  | Women | 4.3% | 21.7% | 34.8% | 39.1% |
| During contraceptive counseling. it can be helpful to know how long the conversation will last. | HCPs | 3.2% | 12.9% | 9.7% | 74.2% |
|  | Women | 0.0% | 13.0% | 8.7% | 78.3% |
| During contraceptive counseling. it can be helpful to have the option to divide the counseling into two or more shorter sessions. | HCPs | 6.5% | 22.6% | 29.0% | 41.9% |
|  | Women | 26.1% | 39.1% | 4.3% | 30.4% |
